# Total levator aponeurosis resection for primary congenital ptosis with very poor levator function

**DOI:** 10.4103/0974-620X.71886

**Published:** 2010

**Authors:** Abdullah Al-Mujaini, Upender K. Wali

**Affiliations:** Department of Ophthalmology, College of Medicine and Health Science, Sultan Qaboos University, Muscat, Sultanate Oman

**Keywords:** Congenital ptosis, levator aponeurosis, Whitnall

## Abstract

**Aim:**

This study was designed to evaluate the effectiveness of total levator aponeurosis resection in patients with very poor levator function secondary to primary congenital ptosis.

**Design:**

A retrospective, noncomparative single-institutional study was designed.

**Participants:**

Seven patients with very poor levator function secondary to primary congenital ptosis operated between May 2008 and May 2010 by one surgeon (AM).

**Materials and Methods:**

A retrospective study of seven patients with congenital ptosis evaluating eyelid elevation following total levator aponeurosis resection. End result is improvement of the eyelid elevation.

**Conclusion:**

Total levator aponeurosis resection is easy and effective tool in elevating the eyelid in patients with very poor levator function secondary to primary congenital ptosis.

## Introduction

Ptosis is an abnormal low position of the upper eyelid which may be congenital or acquired. Primary congenital ptosis is present at birth and tends to be nonprogressive. It may be bilateral, isolated, or part of an associated syndrome. There is harmony between its severity and levator function. Commonly, it is due to the poor development of the levator muscle or its replacement by fibrosis, fat, or areolar tissue.[1] Amblyopia is rare in congenital ptosis unless it is associated with severe unilateral ptosis, anisometropia, or strabismus.[2] Anatomically ptosis may be classified as neurogenic (third nerve palsy, Horner syndrome, and Marcus Gunn Jaw-winking syndrome), myogenic (myasthenia gravis, myotonic dystrophy, ocular myopathy, simple congenital, or blepharophimosis syndrome), aponeurotic (involutional, postoperative), and mechanical (dermatochalasis, tumors, edema, anterior orbital lesions, and scarring).[3]

## Materials and Methods

**Figure 1:**
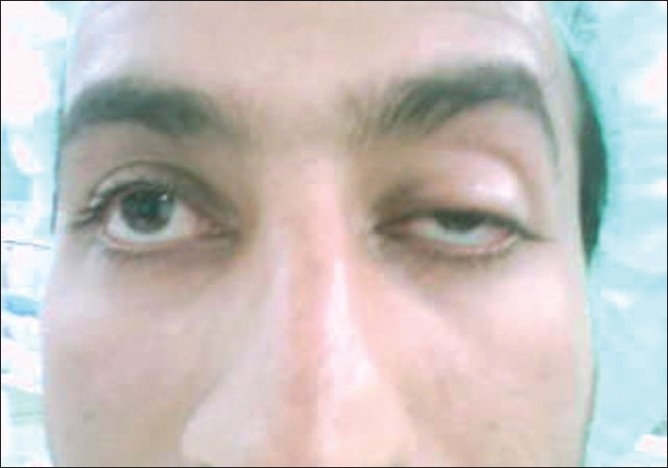
Preoperative findings of the left eye showing; over-elevation of the eyebrow, absence of lid crease, margin reflex distance of -1, and very poor levator function

**Figure 2:**
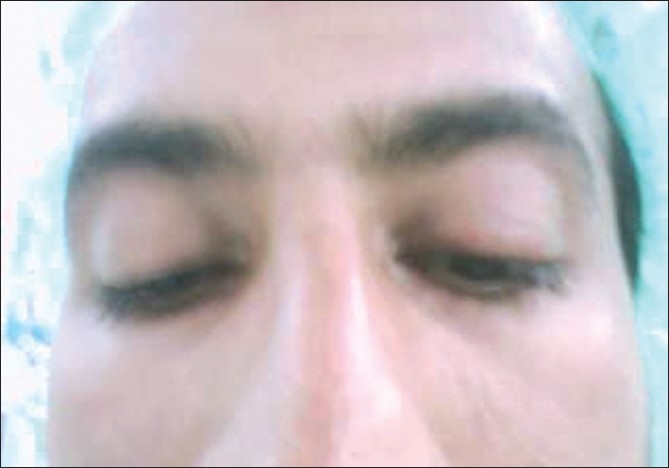
Preoperative findings of the same eye showing lagophthalmos in downgaze

In this retrospective, interventional, single-institutional study seven patients having unilateral primary congenital ptosis with very poor levator function (<5 mm) were enrolled to determine the effectiveness of total levator aponeurosis resection in elevating the eyelid position. All patients were admitted through eye clinic at the university hospital over 2 years from May 2008 to May 2010. The patients were identified from the electronic medical record (EMR). A complete medical history was obtained. All patients underwent testing for the best-corrected visual acuity. No amblyopia was detected in any of them. Detailed ocular examination was carried out for extraocular movements, pupillary reaction, corneal sensation, exposure keratopathy, lagophthalmos, Bell’s phenomenon, and synkinesis such as the presence of Marcus Gunn Jaw-winking ptosis. Physical evaluation included recording of vertical fissure height, margin reflex distance, upper lid crease position, and assessing levator function [Figures 1 and 2]. It also included checking head position, chin elevation, brow position, and brow action in attempted upgaze. All the patients had detailed systemic evaluation to rule out secondary cause of the ptosis.

### Surgical technique

**Figure 3:**
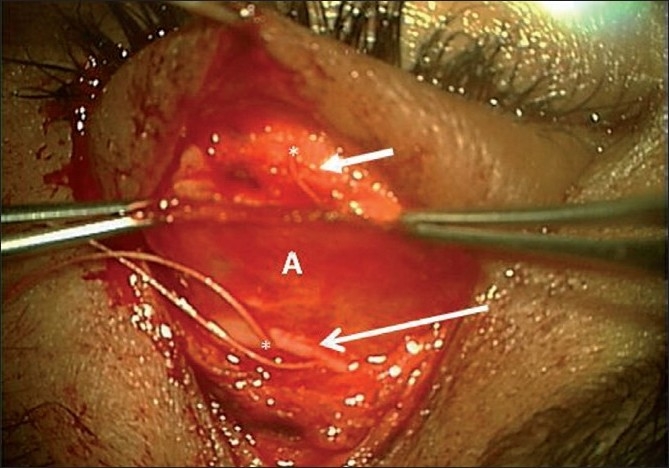
Intraoperative findings of the same patient showing levator aponeurosis dissected and freed completely from the tarsal plate till Whitnall’s (A), tarsal plate (short arrow), Whitnall’s ligament (long arrow), and sutures placed at tarsal plate and retrieved all the way from Whitnall’s beneath the levator aponeurosis (*)

Five patients were operated under general anesthesia and remaining two under local anesthesia using lignocaine 2% and light sedation (midazolam, 1 mg). After preparing and draping, an incision was marked at a level symmetric with the opposite eyelid usually 8–10 mm above the lid margin. A cut was made along the marked line using #15 scalpel blade. The postorbicular fascial plane and the orbital septum were entered, and the attachments between the septum and aponeurosis were separated to prevent postoperative lagophthalmos. The aponeurosis and Whitnall’s ligament were revealed by brushing the preaponeurotic fat pockets upward. This was followed by disinsertion of the aponeurosis from the tarsus. Carrying blunt dissection, the muscle was dissected all the way to the Whitnall’s ligament. A 5.0 vicryl was passed through partial thickness of the tarsus, 3 mm from its upper border and above the central pupil posterior to the aponeurosis and retrieved through the Whitnall’s [Figure 3]. Two additional sutures were added between the tarsus and Whitnall’s and placed medially and laterally. The three sutures were adjusted as needed. Finally, the skin incision was closed with running 6.0 vicryl suture.

## Results

**Figure 4:**
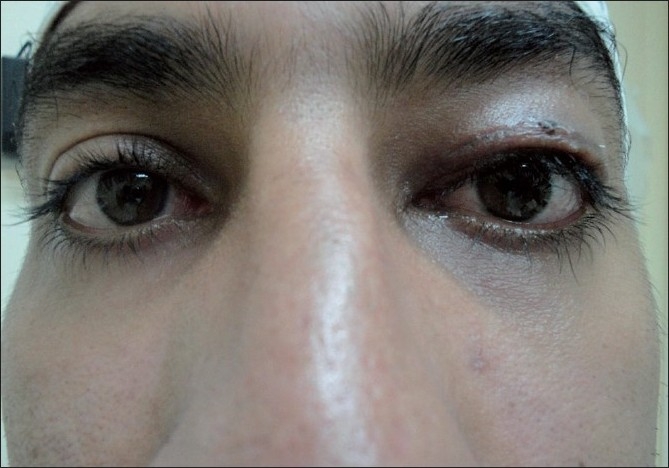
Two months postoperative of the same patient showing dramatic improvement of MRD +3 and good opening of the plapebral fissure

**Figure 5:**
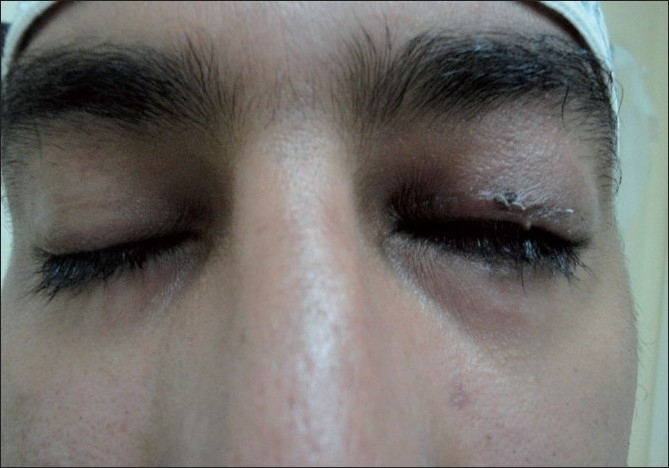
Postoperative finding showing ability of the patient to close his eye

Seven patients having unilateral primary congenital ptosis with very poor levator function underwent total levator aponeurosis resection. The patients included five females and two males. Their ages were ranging from 16 to 32 years. The preoperative and postoperative physical evaluation included recording of vertical fissure height, margin reflex distance, upper lid crease position, and assessing levator function were compared [Tables 1 and 2]. The follow-up period ranged from 2 months to 2 years. At the end of the follow-up period, a dramatic improvement was noted in the lid position, contour, and height in all patients [Figures 4 and 5].

**Table 1:** Preoperative physical evaluation of the ptotic upper eyelid

*Pt no.*	*Gender*	*Age*	*LF, mm*	*MRD, mm*	*VPFH, mm*	*LC*
1.	F	16	3–4	0.0	6	Absent
2.	F	17	4–5	1.0	7	Absent
3.	F	28	4–5	1.0	7	Absent
4.	M	23	3–4	0.0	6	Absent
5.	F	20	3–4	0.0	6	Absent
6.	M	32	1–2	−1.0	5	Absent
7.	F	22	4–5	1.0	7	Absent

LF: Levator function; MRD: Margin reflex distance; VPFH: Vertical palpebral fissure height; LD: Lid crease

**Table 2:** Postoperative improvement of MRD and VPFH

*Patient no.*	*RD, mm*	*PFH, mm*
1.	2–3	8–9
2.	3	10
3.	3	10
4.	3–4	9–10
5.	3	9
6.	2–3	7–8
*7.*	*3*	*10*

MRD: margin reflex distance; VPFH: vertical palpebral fissure h eight

## Discussion

More than 100 techniques for the treatment of ptosis have been reported.[4–6] This means ptosis is difficult to treat, as the postoperative eyelid position may be unpredictable.

Different surgical techniques have been laid out for the management of primary congenital ptosis.[7] This depends upon severity of ptosis, laterality, and levator function. The surgical approach may include posterior resection for mild ptosis with normal levator function or levator aponeurosis resection for moderate-to-poor levator function, and frontalis suspension for bilateral ptosis with poor to absent levator function.[8] In our patients, a modified Whitnall’s sling procedure or total levator aponeurosis resection has given the best results with excellent patient satisfaction despite the fact that the levator function was extremely poor (<5 mm). Although it has been reported that extra-large levator resection may lead to lagophthalmos, none of our patients has experienced this complication. The lagophthalmos may not be a problem as it depends on the orbicularis tone and function. Every ptosis surgery has goals such as controlled height, contour, lid crease, lash position, and symmetry. We found that our patients achieved almost all such targets.

Ptosis can have a marked impact on a patient’s functional status[9] and lead to poor visual development in childhood with its damaging social and financial consequences in later life.[2] The goal of ptosis surgery was once described as one with elusive result.[10] Ptosis surgery in pediatric patients’ differed from adult surgery in that predictability of lid height in later group could be enhanced by using local anesthesia or adjustable sutures.[11,12] As there were no authentic published data regarding time taken to reach final lid height stability in primary congenital ptosis patients, we chose a maximum follow-up of 2 years as a stable end point.

Ptosis surgery carries risk of complications such as corneal exposure, conjunctival prolapse and abnormality of lid crease, and contour.[13] None of our patients had any of these complications which proves the efficacy of this technique in our hands. Anderson *et al*. reported an incidence of 30.7% of undercorrection over a 1-year follow-up in a series of 69 ptotic eyelids that were corrected with maximum levator aponeurosis resection (Whitnall’s sling) without tarsectomy. In our small group, we did not report any incidence of such complication over 2 years follow-up period.[14]

## Conclusion

In this series, the authors describe seven patients with primary congenital ptosis and extremely poor levator function treated successfully with total levator aponeurosis resection technique instead of frontalis suspension which is the recommended surgery in these circumstances.[5] All the patients have achieved the desire result without any complications. Although recent findings have shown the frontalis suspension technique is a commonly performed surgical correction of congenital ptosis, used widely in the repair of ptosis with poor levator function, we recommend ours as an ideal procedure to start with for the correction of primary congenital ptosis with very poor levator function.
